# Caregiver Nutrition and Nurturing Care: A Scoping Review

**DOI:** 10.1111/mcn.70058

**Published:** 2025-06-16

**Authors:** Taryn J. Smith, Alice Fortune, Melissa J. Gladstone

**Affiliations:** ^1^ Department of Women's and Children's Health Institute of Life Course and Medical Sciences, University of Liverpool UK

**Keywords:** anaemia, anthropometry, diet, early childhood development, early learning, micronutrients, psychosocial stimulation, responsive caregiving

## Abstract

Research on early childhood development has focused on child health, nutrition and stimulation. However, less attention has been given to the role of caregiver nutrition in shaping caregiving behaviours. Suboptimal caregiver nutrition may impair the ability to provide responsive and nurturing care. This scoping review aimed to summarise the existing evidence on the link between caregiver nutrition and nurturing care, specifically responsive caregiving and early learning opportunities. Database (Medline) and citation searches yielded 23 articles meeting inclusion criteria (*n* = 17 observational; *n* = 6 randomised controlled trials [RCTs]). The majority (*n* = 15) were conducted in low‐ and middle‐income countries. Observational studies measured caregiver anthropometry (*n* = 8), dietary intakes/diversity/quality (*n* = 6), anaemia (*n* = 6) and vitamin B6 status (*n* = 1). RCTs supplemented pregnant and/or postpartum women with iron (*n* = 2), multiple micronutrients (*n* = 2), fish oil (*n* = 1) and food‐based snacks (*n* = 1). Most articles (*n* = 18) measured caregiving through live or videotaped observations of caregiver–child interactions; the remaining used caregiver self‐reported measures of stimulation or caregiver–child bonding/relationship. Overall, suboptimal diets, food insecurity, caregiver under‐ and overnutrition, anaemia and low vitamin B6 status were associated with less responsive caregiving and fewer opportunities for early learning. Providing anaemic or food‐insecure caregivers with iron or food‐based supplements positively altered caregiver–child interactions. Supplementation trials that did not specifically target undernourished caregivers found no effects on caregiving behaviours. More research specifically targeting undernourished caregivers is needed to understand how nutritional interventions might benefit caregiving. Interventions aimed at enhancing nurturing care should consider both caregiver and child nutrition as potential targets to improve outcomes for both children and their caregivers.

## Summary

- Suboptimal caregiver dietary intakes, food insecurity, caregiver under‐ and overnutrition and anaemia were negatively associated with responsive caregiving and early learning in observational studies.
- The limited intervention trials conducted to date revealed inconsistent findings but suggest that nutritional interventions targeting undernourished caregivers could potentially have a positive impact on caregiving behaviours.
- Evidence for micronutrients other than anaemia/iron deficiency is limited.
- Further research is required, particularly intervention trials targeting undernourished caregivers and considering a wider range of macro‐ and micronutrients.
- Multicomponent interventions combining nutritional support for caregivers and children with behavioural recommendations for responsive care and stimulation may benefit child development.

## Graphical abstract

Suboptimal caregiver diets, food insecurity, caregiver under‐ and overnutrition, anaemia and low vitamin B6 status were associated with less responsive caregiving and fewer opportunities for early learning. Interventions aimed at enhancing nurturing care should consider both caregiver and child nutrition as potential targets to improve outcomes for both children and their caregivers.

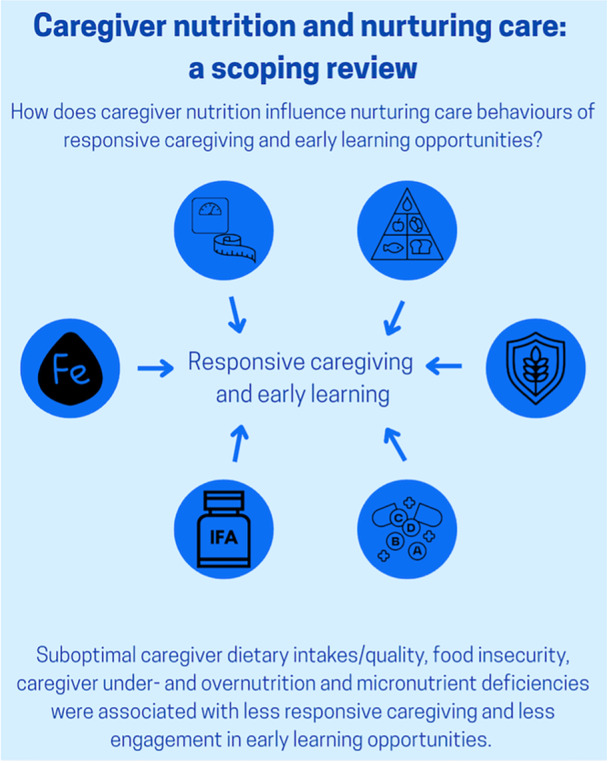

## 1 Introduction

Nurturing care is crucial in facilitating children's attainment of their full developmental potential. Nurturing care refers to stable and secure environments that support multiple aspects of early childhood development (Britto et al. 2017). This concept is underpinned by the Nurturing Care Framework and its five inter‐related components of health, nutrition, responsive caregiving, early learning opportunities and safety and security (World Health Organization, United Nations Children's Fund, & World Bank Group 2018).

Responsive caregiving and caregivers' engagement in early learning have been identified as key caregiving behaviours that support children's cognitive, motor, language and socioemotional development across diverse populations and settings (Jeong et al. 2021). Responsive caregiving refers to caregivers' responses to children's cues that are prompt, contingent on children's behaviour, emotionally supportive and developmentally appropriate (Black and Aboud 2011). This fosters secure attachments and self‐regulation, which facilitate exploration and learning, the ability to manage emotions and exercise control over impulses – crucial aspects of socioemotional development (Dunst and Kassow 2008; Cabral de Mello 2006). Caregivers' engagement in early learning activities such as reading, storytelling, playing, singing, and naming, counting or drawing objects, promotes children's cognitive, language and socioemotional development (Hamadani et al. 2010; Jeong et al. 2017). These experiences predict not only children's early development but also their longer‐term educational, economic, physical and mental health outcomes (Gertler et al. 2014; Walker et al. 2006; Walker et al. 2011).

Caregiver–child interactions are particularly influential during the period of rapid brain development spanning the first 2 years of life when young children spend extended periods of time with their main caregivers (primarily mothers, but also fathers, grandparents and others) in their immediate environment (i.e., the home) (Grantham‐McGregor et al. 2007). Higher maternal education, having fewer children, household ownership of books/toys, greater household socioeconomic status and food security have been positively associated with more responsive and sensitive caregiver–child interactions and greater stimulation practices within the home (Kim et al. 2021; Obradović et al. 2016; Scherer et al. 2019). Conversely, poor caregiver mental health has been associated with negative parenting behaviours and lower responsive caregiving (Huang et al. 2017; Scherer et al. 2019). Consequently, parenting interventions and home visiting counselling packages have increasingly focused on supporting caregivers and particularly their mental health and well‐being (Kim et al. 2021; Pitchik et al. 2021; Singla et al. 2015; Tofail et al. 2023; United Nations Children's Fund 2024).

Parenting programmes have traditionally focused on child development (e.g., stimulation, play, communication), often alongside child nutrition interventions (Grantham‐McGregor et al. 2014), with a few recent programmes also incorporating caregiver mental health. However, much less attention has been given to the nutritional status of caregivers, despite the Nurturing Care Framework emphasising the equal importance of both caregiver and child nutrition for optimal early childhood development. In simple terms, we cannot expect caregivers to provide the best care for their children if their own health and nutrition are not prioritised. Poor caregiver nutrition, such as micronutrient deficiencies, leads to reductions in energy, cognition, mood and overall capacity, directly impacting their ability to provide optimal care (Prado et al. 2012). Globally, women lack access to healthy diets and in 2015, 14.2% of women 20–49 years of age living in low‐ and middle‐income countries (LMICs) had a low body mass index (BMI) (Victora et al. 2021). Furthermore, it is estimated that 69% of nonpregnant women of reproductive age (15–49 years) globally suffer from at least one micronutrient deficiency, with the highest prevalence in sub‐Saharan Africa (80%) and South Asia (74%) (Stevens et al. 2022). Adequate caregiver nutrition is a fundamental resource that enables caregivers to provide the nurturing care essential for optimal child health, nutrition and development (Engle et al. 1999). Children have the right to develop to their full potential (United Nations 1989) and without ensuring caregivers are well‐nourished and in good health, even the best child‐focused interventions will fall short.

To the best of our knowledge, no review to date has bought together observational or trial evidence on caregiver nutrition and responsive caregiving or engagement in early learning practices. Given the rapidly growing interest by researchers and policymakers in parenting interventions targeting caregivers of infants and young children to optimise child development, a scoping review summarising the available evidence on caregiver nutrition and caregiving behaviours may help to uncover potential additional pathways that benefit early childhood development through improved caregiving behaviours. The aim of this scoping review was to synthesise the available evidence on associations between caregiver nutrition and responsive caregiving and early learning, as well as interventions targeting caregiver nutrition through macronutrient/micronutrient supplementation, to improve caregiving behaviours.

## 2 Methods

### 2.1 Search Strategy

Scoping reviews are useful for summarising existing evidence in research areas that have yet to be reviewed, and for exploring key concepts and identifying knowledge gaps and further research needs (Arksey and O'Malley 2005). This scoping review is reported according to the Preferred Reporting Items for Systematic Reviews and Meta‐Analysis (PRISMA) extension for scoping reviews (Tricco et al. 2018). A comprehensive search of Medline (Ovid) was completed in July 2024 using search terms specific to the population group of interest, nutrition exposures and caregiving outcomes (Table S1). Additional relevant articles were identified through citation searching, by reviewing reference lists of the included articles, as well as identifying more recent articles that have cited the included articles.

### 2.2 Eligibility Criteria

Published observational and experimental studies were eligible if they included (1) pregnant women, postpartum women or mother/caregiver–child dyads where the child was < 5 years of age, (2) any measure of caregiver nutrition, including anthropometry, biomarkers of nutritional status, dietary intakes or indicators (e.g., dietary diversity) or any intervention that provided caregivers with macro‐ or micronutrient supplements or food supplements and (3) outcome measures related to nurturing care components of responsive caregiving and early learning, including caregiver–child interactions/relationships and stimulation (Table S2). No restrictions were placed on the geographic location of studies or publication years (database inception through July 2024 included). Only articles reported in English were included.

### 2.3 Screening, Data Extraction and Synthesis of Results

Search results were imported into Covidence (Veritas Health Innovation, Melbourne, Australia). Two reviewers (T.J.S. and A.F.) independently screened article titles and abstracts based on the criteria outlined above. Disagreements were resolved through discussion. One reviewer (T.J.S.) conducted a full‐text review and extracted data from the included articles into a standardised template. Extracted data included authors, publication year, study design, country, data collection years, population group, sample size, measure of maternal nutrition, outcome measure and the tool used, and the main findings. For intervention studies, the specific intervention, the timing of the intervention and the duration were also extracted. The extraction sheet was reviewed and confirmed by a second reviewer (A.F.). As per scoping review methods, a quality appraisal was not conducted (Arksey and O'Malley 2005).

Results were summarised narratively by study design (observational and intervention) and caregiver nutrition exposure (dietary intakes/diversity/quality, anthropometry, anaemia/iron status and interventions).

## 3 Results

### 3.1 Characteristics of Included Articles

**Figure 1:**
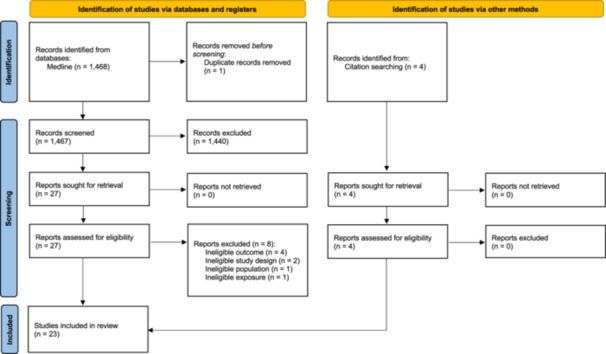
PRISMA flow diagram of search results and included articles.

A total of 1467 records were screened by title and abstract. Of these, 27 articles were included for full‐text review, of which 8 were subsequently excluded and 4 additional articles were identified through citation searching (Figure 1). A total of 23 articles were included in the final review, reporting on 17 unique studies.

Among the included articles, 9 had a longitudinal design, 6 a cross‐sectional design and 2 were a cross‐sectional analysis of baseline data from cluster randomised controlled trials (Table 1). Six articles were randomised controlled trials (RCTs; from 4 unique studies) (Table 2). Most of the articles (*n* = 15) reported on studies that were conducted in LMICs, specifically Bangladesh (*n* = 5), Egypt, Kenya and Malawi (*n* = 3 each), South Africa (*n* = 2) and Vietnam (*n* = 1) (some studies were conducted in more than one country). Articles reporting on findings from high‐income countries (HICs; *n* = 8) were conducted in Germany and the USA (*n* = 2 each), Canada, Chile, Uruguay and the UK (*n* = 1 each). Articles were published between 1990 and 2024, with data collected between 1984 and 2022, although 8 articles did not specify data collection years. Sample sizes in observational studies ranged from 7 to 8429, and in interventions between 66 and 669. Studies primarily included mothers, but three studies included other caregivers (e.g., grandmothers).

**Table 1:** Study characteristics and main findings of observational studies and analyses.

Author (year)	Study characteristics (study design, country (sample size), population)	Caregiver nutrition measure	Outcomes assessed	Outcome assessment tool	Main findings
**Caregiver dietary intakes/diversity/quality**					
Wachs et al. (1992)	Study design: longitudinal Country (sample size): Kenya (*n* = 110) and Egypt (*n* = 153) Population: caregiver–child dyads (children 18–30 months of age)	Dietary intake: weighed food recall (2 consecutive days each month over a 12‐month period)	Caregiver–child interaction	Setting: home observation; naturalistic interaction during daily activities. Observation period: Kenya – 120 min. Egypt – 30 min. Coding scheme: researcher developed for the study based on previous African and Western studies. Kenya – 1 min time sampling procedure used (30 s for observations and 30 s for recording). Behaviours that occurred ≥ 1 time in a specific 30 s interval were scored only once. Egypt – direct coding (i.e., every time a specific behaviour occurred it was recorded). Behaviours coded: percentage of child vocalisations responded to either verbally or non‐verbally; number of spontaneous vocalisations by the caregiver to the child; percentage of time there was no response to child's distress; total number of times child was picked up or held by caregiver; speed of caregiver response to child distress (Egypt only).	Kenya: Caregivers with greater dietary intakes demonstrated less physical contact with their child. Egypt: Caregivers with greater dietary intakes vocalised more to their children, provided fewer non‐verbal responses to their children's vocalisations, but were slower to respond to their child's distress signals.
Rahmanifar et al. (1993)	Study design: longitudinal Country (sample size): Egypt (*n* = 41) Population: caregiver–child dyads (children 3–6 months of age)	Dietary intake: 24 h recall (2 consecutive days each month) Haemoglobin levels	Caregiver–child interaction	Setting: home observation; naturalistic interaction during daily activities. Observation period: 30 min. Coding scheme: not specified. Direct coding was utilised (i.e., every time a specific behaviour occurred it was recorded). Behaviours coded: number of spontaneous vocalisations of the infant; number of caregiver vocalisations to the infant; number and duration of physical contact with the infant; number and duration of episodes of infant distress; caregiver's verbal responsiveness to infant's vocalisations; caregiver's response to infant distress.	Poorer diet quality and lower caregiver haemoglobin levels were associated with less response to infant vocalisations.
McDonald et al. (1994)	Study design: longitudinal Country (sample size): Kenya (*n* = 110) Population: caregiver–child dyads (children 18–30 months of age)	Dietary intake: weighed food intake and food recall (2 consecutive days each month)	Caregiver–child interaction	Setting: home observation; naturalistic interaction during daily activities. Observation period: 90–120 min. Coding scheme: not specified. 1 min time sampling procedure used (30 s for observations and 30 s for recording). Behaviours coded: cares for physical needs; holds/carries; touches; interacts socially; talks to child; responds to child vocalisations (touch and interacts socially was later dropped from analyses due to infrequent behaviours).	During a 3.5‐month food shortage, caregivers spent less time caring for, holding and talking to their children, and this was greatest for the poorest families.
Mortaji et al. (2021)	Study design: longitudinal Country (sample size): Canada (*n* = 808) Population: caregiver–child dyads (children 3–4 years of age)	Maternal dietary quality (Healthy Eating Index 2010) during pregnancy (16–21 weeks gestation)	Stimulation and caregiver interactions within the home	Setting: home; interview and home observation. Tool: HOME Inventory. Behaviours coded: caregiver responsivity, acceptance and involvement, learning materials, variety and organisation in the child's environment.	A statistically significant interaction was observed between maternal diet quality during pregnancy and HOME scores on measures of child executive functioning, suggesting that increased maternal diet quality during pregnancy was associated with better executive functioning in less stimulating home environments.
Bliznashka et al. (2024)	Study design: cross‐sectional analysis of a cluster RCT Country (sample size): Malawi (*n* = 1021) Population: caregiver–child dyads (children < 2 years of age)	Minimum dietary diversity for women	Stimulation within the home	Setting: self‐report. Tool: Family Care Indicators. Behaviours coded: the number of times caregivers engaged in 14 types of stimulation activities with their child in the preceding 3 days.	Greater caregiver dietary diversity was directly associated with a higher stimulation score.
**Caregiver anthropometry**					
Valenzuela (1997)	Study design: cross‐sectional Country (sample size): Chile (*n* = 85) Population: caregiver–child dyads (children ~18 months of age) (*n* = 42 underweight children and *n* = 43 normal weight children)	Weight	Caregiver sensitivity	Setting: home observation; naturalistic interaction during daily activities. Observation period: 4 h. Coding scheme: Maternal Sensitivity Scale. Behaviours coded: sensitivity scored on a scale of 1 – 9 based on observations of the caregiver's ability to accurately perceive and promptly respond to the child's signals. Higher scores represent more sensitive behaviour. Unresponsiveness or delays in responding to the child's signals receive lower scores.	Caregiver weight was significantly positively correlated with sensitivity, independent of socioeconomic variables (income, household size, number of children in the household, mother's and father's age, father's occupation).
Rising and Lifshitz (2005)	Study design: cross‐sectional Country (sample size): USA (*n* = 7) Population: caregiver–child dyads (children ~5 months of age)	BMI (*n* = 4 caregivers with obesity and *n* = 3 with normal weight) Body composition	Caregiver–child interaction time	Setting: research laboratory. Observation period: 24 h. Coding scheme: research developed for study. Behaviours coded: interaction time and type over 24 h. Interaction time was calculated as the sum of all interaction periods over the 24‐h observation. Interactions recorded included feeding, holding, cuddling and nappy/diaper changes.	Caregivers with obesity spent less time interacting with their infants over the 24‐h observation period than caregivers with normal weight (381 min vs. 570 min). There was a negative correlation between total 24‐h interaction time and caregiver body weight and body fat.
Hamadani et al. (2012)	Study design: longitudinal Country (sample size): Bangladesh (*n* = 488) Population: mothers at 6–9 weeks postpartum	BMI MUAC Anaemia (Hb < 11 g/dL)	Stimulation and caregiver interactions within the home	Setting: home; interview and home observation. Tool: HOME Inventory (Infant‐Toddler version) adapted to the local context. Behaviours coded: caregiver responsivity, acceptance and involvement, learning materials, variety and organisation in the child's environment.	Maternal BMI and MUAC were significantly positively correlated with maternal caregiving scores. There was no relationship between maternal anaemia and caregiving scores.
Keitel‐Korndörfer et al. (2015)	Study design: longitudinal Country (sample size): Germany (*n* = 62) Population: caregiver–child dyads (children 19–58 months of age)	BMI (*n* = 31 caregivers with obesity and *n* = 31 with normal weight)	Caregiver–child attachment	Setting: videotaped at home; naturalistic interaction during daily activities. Observation period: 2 h. Coding scheme: Attachment Q‐Set. Behaviours coded: 90 items of caregiver–child interaction and child behaviour coded. A higher score indicates the child's ability to use the caregiver as a secure base.	The quality of the caregiver–child attachment was lower among the group of caregivers with obesity compared to caregivers with normal weight. Indicates that children of caregivers with obesity are less likely to use their caregivers as a secure base.
Bergmann et al. (2016)	Study design: longitudinal Country (sample size): Germany (*n* = 146) Population: caregiver–child dyads (children 6–47 months of age)	BMI (*n* = 73 caregivers with obesity and *n* = 73 with normal weight)	Caregiver–child interaction	Setting: videotaped in a research laboratory; interactions during free play with age‐appropriate toys. Observation period: 16 min. Coding scheme: Emotional Availability Scale. Coded behaviours: four caregiver scales – sensitivity, structuring, non‐intrusiveness and non‐hostility; two child scales – responsiveness and involvement.	Caregivers with obesity had significantly lower scores on all four adult dimensions than caregivers with normal weight. Child dimension scores did not differ. Total emotional availability score was significantly lower for caregivers with obesity and their children than caregivers with normal weight.
Basnet et al. (2020)	Study design: cross‐sectional Country (sample size): Bangladesh (*n* = 4400) and Vietnam (*n* = 4029) Population: caregiver–child dyads (children < 5 years of age)	Height BMI	Stimulation within the home	Setting: self‐report. Tool: Family Care Indicators. Behaviours coded: number of stimulating activities which an adult engaged in with child in the previous 3 days. Activities were: reading books; telling stories; singing songs; taking outside; playing; naming, counting and drawing.	Caregiver height was positively associated with stimulation practices among older children (24–59.9 months) in Vietnam. Caregiver BMI was positively associated with stimulation practices among older children (24–59.9 months) in Bangladesh. No associations between caregiver height or BMI and stimulation practices among younger children (6–23.9 months of age).
Daniel et al. (2021)	Study design: cross‐sectional analysis of a cluster RCT Country (sample size): Malawi (*n* = 85) Population: children with severe acute malnutrition and their caregivers (children 6–59 months of age)	BMI	Stimulation and caregiver interactions within the home	Setting: home; interview and home observation. Tool: HOME Inventory adapted to the local context. Behaviours coded: caregiver responsivity, acceptance and involvement, learning materials, variety and organisation in the child's environment.	Caregiver BMI was predictive of better HOME scores (i.e., more stimulating home environment and more caregiver interactions with child).
**Caregiver anaemia/iron status**					
Azizi‐Egrari et al. (2004)	Study design: longitudinal Country (sample size): Kenya (*n* = 124) Population: caregiver–child dyads (children 2–6 months of age)	Anaemia (Hb < 12.5 g/dL) Dietary intake: weighed food recall (2 consecutive days each month) BMI	Caregiver–child interaction	Setting: home observation; naturalistic interaction during daily activities. Observation period: 120 min. Coding scheme: researcher developed for study based on previous African and Western studies. 1 min time sampling procedure used (30 s for observations and 30 s for recording). Any number of behaviours could be coded in one time frame. Behaviours coded: physical care (e.g., feeding, washing, changing clothes, putting to sleep); holding/carrying; touching; face‐to‐face contact or looking at each other; caregiver talking to the child.	Anaemic caregivers spent less time holding and caring for their infants than non‐anaemic caregivers. Caregiver dietary intakes, weight and BMI were not associated with any caregiving behaviours.
Kordas et al. (2011)	Study design: cross‐sectional Country (sample size): Uruguay (*n* = 109) Population: caregiver–child dyads (children 15–55 months of age)	Anaemia (Hb < 12 g/dL)	Stimulation and caregiver interactions within the home Caregiver perception of parenting/relationship with child	Setting: self‐report. Tools: HOME Inventory (interview items only). Self‐Efficacy for Parenting Tasks Index ‐ Toddler Scale. Behaviours coded: HOME Inventory ‐ caregiver responsivity, acceptance and involvement, learning materials, variety and organisation in the child's environment. Self‐Efficacy for Parenting Tasks Index – caregivers were given 19 statements describing various ways in which parents and children may relate to one another. Caregivers were asked to determine whether those statements applied to their relationship with their child. Domains assessed: emotional availability; teaching; structure; nurturance; discipline.	Caregiver anaemia was associated with a lower likelihood that caregivers would give their children opportunities to explore and play.
Aubuchon‐Endsley et al. (2012)	Study design: cross‐sectional Country (sample size): USA (*n* = 105) Population: mothers at 3 months postpartum	Iron status (haemoglobin, soluble transferrin receptor, ferritin)	Parenting style	Setting: self‐report. Tool: Parenting Styles and Dimensions Questionnaire. Behaviours coded: mothers responded to statements about the frequency of parenting practices within the home on a 5‐point Likert scale. Yields three scale scores for parenting styles: authoritative, authoritarian, permissive.	Mothers with haemoglobin levels < 14.0 g/dL, depressive symptomatology was positively associated with authoritarian parenting style (verbal hostility, corporal punishment, non‐reasoning, directiveness), which may manifest as insufficient emotional support and nurturing in mother–child relationships.
Dearman et al. (2012)	Study design: case–control Country (sample size): UK (*n* = 115) Population: mothers within 4 weeks of delivery	Anaemia (Hb < 10 g/dL) (*n* = 57 anaemic and *n* = 58 non‐anaemic)	Mother–infant bonding	Setting: self‐report. Tool: Postpartum Bonding Questionnaire. Behaviours coded: impaired bonding; rejection and pathological anger; infant‐focused anxiety.	No statistical difference in maternal perception of mother–infant bonding between anaemic and non‐anaemic groups.
**Caregiver vitamin B6 status**					
McCullough et al. (1990)	Study design: longitudinal Country (sample size): Egypt (*n* = 69) Population: caregiver–child dyads (children 3–6 months of age)	Breastmilk vitamin B6 concentration as an indicator of maternal vitamin B6 status	Caregiver–child interaction	Setting: home observation; naturalistic interaction during daily activities. Observation period: 30 min. Coding scheme: researcher developed for study based on previous African and Western studies. Behaviours coded: amount and duration of physical contact; carrying while doing other activities; number of spontaneous vocalisations from caregiver to child; verbal responsivity to child's vocalisations; if caregivers' response was effective in soothing child; no response to child distress.	Poor maternal vitamin B6 status (breastmilk B6 concentration < 430 nmol/L) was associated with higher levels of nonresponse to infant vocalisation, less effective intervention to infant distress and less time spent on childcare.

Abbreviations: BMI, body mass index; Hb, haemoglobin; HOME Inventory, Home Observation Measurement of the Environment; MUAC, mid‐upper arm circumference; RCT, randomised controlled trial.

**Table 2:** Study characteristics and main findings of randomised controlled trials.

Author (year)	Country (sample size)	Caregiver nutrition measure	Intervention	Outcomes assessed	Outcome assessment tool	Main findings
**Iron supplementation**						
Perez et al. (2005)	South Africa (*n* = 81)	Iron deficiency anaemia	At 10 weeks postpartum, women were randomised to one of three interventions until 9 months postpartum:	Mother–child interaction (at child age 10 weeks and 9 months)	Setting: videotaped in a health clinic; interactions during free play. Observation period: 20 min. Coding scheme: Parent/Caregiver Involvement Scale (PCIS). Behaviours coded: 11 scales that assess physical and verbal interaction, responsiveness, play, teaching, control of activities, directives‐demands, relationship, positive and negative statements and goal setting. For each scale, the amount, quality and appropriateness were determined and assigned a score (1–5).	At baseline (10 weeks), anaemic mothers provided fewer positive and negative statements, were less responsive, and exerted more control over their infant's behaviours. At follow‐up (9 months), anaemic mothers in the IDA‐PL group had more negative statements and were less responsive than mothers in the control group. In contrast, the behaviours of anaemic mothers treated with iron were similar to that of control mothers on all 11 scales of the PCIS.
			IDA‐Fe (*n* = 30): iron‐deficient anaemic mothers received iron (125 mg) + vitamin C (25 mg) + folic acid (10 µg) daily. IDA‐PL (*n* = 21): iron‐deficient anaemic mothers received a placebo of vitamin C (25 mg) + folic acid (10 µg) daily. Non‐anaemic control mothers (*n* = 30) received no supplement.			
Murray‐Kolb and Beard (2009)	South Africa (*n* = 66)	Iron deficiency anaemia	At 10 weeks postpartum, women were randomised to one of three interventions until 9 months postpartum:	Mother–child interaction (at child age 10 weeks and 9 months)	Setting: videotaped in a health clinic; interactions during free play. Observation period: 20 min. Coding scheme: Emotional Availability Scale. Behaviours coded: four caregiver scales – sensitivity, structuring, non‐intrusiveness and non‐hostility; two child scales – responsiveness and involvement (two child scales only scored at the 9‐month timepoint).	At baseline (10 weeks), the control group of non‐anaemic mothers scored significantly higher on the sensitivity and child responsiveness scales than the two anaemic groups. There was no difference in scale scores between the two anaemic groups (IDA‐PL and IDA‐Fe). At endpoint (9 months), the control and IDA‐Fe groups no longer differed on any of the scales. The mothers in these two groups scored significantly higher than mothers in the IDA‐PL group on the maternal sensitivity, structuring and non‐hostility scales than the IDA‐PL group.
			IDA‐Fe (*n* = 25): iron‐deficient anaemic mothers received iron (125 mg) + vitamin C (25 mg) + folic acid (10 µg) daily. IDA‐PL (*n* = 19): iron‐deficient anaemic mothers received a placebo of vitamin C (25 mg) + folic acid (10 µg) daily. Non‐anaemic control mothers (*n* = 22) received no supplement.			
**Multiple micronutrient supplementation**						
Frith et al. (2009)	Bangladesh (*n* = 180)	Multiple micronutrient supplementation	Pregnant women (14 weeks gestation) randomised to one of three interventions until 3 months postpartum:	Mother–child feeding interaction (children 3–4 months of age)	Setting: home observation; interactions during child feeding. Observation period: all feeding interactions in a single day. Coding scheme: Nursing Child Assessment Satellite Testing Feeding Scale (NCAST). Behaviours coded: four mother subscales – sensitivity, response to infant distress, social emotional growth fostering and cognitive fostering; two infant subscales – clarity of cues and responsiveness to caregiver.	Mothers in the 60 mg Fe group had lower interaction scores than mothers in the 30 mg Fe group, exhibited less positive and more negative interaction behaviours. MMS did not improve interaction behaviours compared to the 30 mg Fe group.
			30 mg Fe (*n* = 58): 30 mg iron + 400 µg folic acid daily. 60 mg Fe (*n* = 67): 60 mg iron + 400 µg folic acid daily. MMS (*n* = 55): multiple micronutrient supplement daily.			
Prado et al. (2018)	Malawi (*n* = 669)	Haemoglobin concentration Vitamin A status (plasma retinol) Breastmilk concentrations of vitamins B1, B2, B3, B6, B12 and DHA	Pregnant women (≤ 20 weeks' gestation) were randomised to one of three groups until 6 months postpartum:	Stimulation and caregiver interactions within the home (at the child's age 6 months)	Setting: home, interview and home observation. Tool: HOME Inventory adapted to the local context. Behaviours coded: caregiver responsivity, acceptance and involvement, learning materials, variety and organisation in the child's environment.	There were no significant differences in HOME scores between the three intervention groups. HOME scores were not associated with maternal nutritional status for any of the biomarkers assessed.
			IFA (*n* = 221): 60 mg iron + 400 µg folic acid daily from enrolment to delivery and placebo containing 200 mg calcium daily from delivery to 6 months postpartum. MMN (*n* = 231): multiple micronutrient supplement daily from enrolment to 6 months postpartum. LNS (217): daily 20 g sachet of a small‐quantity lipid nutrient supplement from enrolment to 6 months postpartum			
**Fish oil supplementation**						
Tofail et al. (2006)	Bangladesh (*n* = 249)	Fish oil or soy oil supplementation	Pregnant women (25 weeks' gestation) were randomised to one of two groups until delivery:	Stimulation and caregiver interactions within the home (at the child's age of 10 months)	Setting: home; interview and home observation. Tool: HOME Inventory adapted to the local context. Behaviours coded: caregiver responsivity, acceptance and involvement, learning materials, variety and organisation in the child's environment.	At the child's age of 10 months, there were no significant differences in HOME scores between the two intervention groups.
			Fish oil capsules (*n* = 125): containing 1.2 g DHA and 1.8 g EPA per day. Soy oil capsules (*n* = 124): containing 2.25 g linoleic acid and 0.27 g α‐linolenic acid per day.			
**Food‐based supplement**						
Frith et al. (2012)	Bangladesh (*n* = 180)	Food insecurity	Pregnant women were randomised to one of two food supplementation groups:	Mother–child feeding interaction (children 3– 4 months of age)	Setting: home observation; interactions during child feeding. Observation period: all feeding interactions in a single day. Coding scheme: Nursing Child Assessment Satellite Testing Feeding Scale (NCAST). Behaviours coded: four mother subscales – sensitivity, response to infant distress, social emotional growth fostering and cognitive fostering; two infant subscales – clarity of cues and responsiveness to caregiver.	In the group that started the food supplement at the usual time (~20 weeks' gestation), food insecurity was associated with reduced quality mother–infant interaction. However, food insecurity was not associated with reduced quality mother–infant interaction in the group assigned to the early food supplement (~9 weeks gestation). The lowest subscale scores (indicating lower quality interaction) were found for the usual start group who were severely food insecure.
			Early invitation start time: daily food supplement (snack: 6 days/week) from ~9 weeks of gestation to delivery. Usual invitation start time: daily food supplement (snack: 6 days/week) from ~20 weeks' gestation to delivery.			

Abbrevations: DHA, docosahexaenoic acid; EPA, eicosapentaenoic acid; HOME Inventory, Home Observation Measurement of the Environment.

Among the 17 articles with an observational design/analysis (Table 1), the most common measure of caregiver nutrition was anthropometry (*n* = 8 articles; including BMI, body weight, height, mid‐upper arm circumference (MUAC) and body composition), followed by dietary intakes, diversity or quality (*n* = 6) and caregiver anaemia or iron status (*n* = 6). One study assessed breastmilk vitamin B6 concentration as an indicator of caregiver vitamin B6 status. Among the 6 articles reporting on RCTs (Table 2), two supplemented anaemic mothers with iron from 10 weeks postpartum to 9 months postpartum. The remaining RCTs supplemented pregnant women with multiple micronutrient supplements (MMS) until 3 months postpartum (*n* = 1), iron‐folic acid, MMS or lipid‐based nutrient supplements until 6 months postpartum (*n* = 1), fish oil supplements until delivery (*n* = 1) or a food‐based supplement until delivery (*n* = 1).

### 3.2 Outcome Assessment Tools and Methodologies

Outcomes assessed and the methods or tools used are described in Tables 1 and 2. Over half (*n* = 13) the articles observed caregiver–child interactions and used coding schemes to quantify caregiver and child behaviours and assess the quality of the interaction observed for a specific period. This was either live coded during the observation (*n* = 9) or later coded from videotaped observations (*n* = 4). Seven of these articles reported observing caregiver–child dyads in a naturalistic manner within the home as they completed their daily activities, while six observed interactions during semi‐structured activities (e.g., free play, feeding) either at home, in a health clinic or in a research laboratory (*n* = 2 for each setting). Seven articles described using existing coding schemes during live or videotaped interactions, including the Emotional Availability Scale (*n* = 2), the Nursing Child Assessment Satellite Testing Feeding Scale (*n* = 2), the Maternal Sensitivity Scale (*n* = 1), the Parent/Caregiver Involvement Scale (*n* = 1) and the Attachment Q‐Set (*n* = 1). Four articles reported using bespoke coding schemes developed for the purpose of the study, while two articles did not specify any coding schemes.

Five articles reported using the Home Observation for the Measurement of the Environment (HOME) Inventory. The HOME Inventory is based on caregiver interview as well as observing caregiver–child interactions in the home. Items assess caregiver responsivity and involvement with the child, provision of appropriate play materials, variety in daily stimulation and organisation of the home environment. Four articles using the HOME Inventory in LMICs reported adapting items to the local context, although only one article (Prado et al. 2018) described these adaptations within the article.

Finally, five articles reported using a variety of caregiver self‐report measures to assess: stimulation practices and opportunities for early learning within the home (e.g., availability of books/toys, play activities with caregivers) using either the Family Care Indicators (FCI; *n* = 2) or the HOME Inventory interview items on their own (without observation) (*n* = 1); caregiver perception of caregiver–child bonding/relationship using the Postpartum Bonding Questionnaire (*n* = 1) and the Self‐Efficacy for Parenting Tasks Index (*n* = 1), and parenting style using the Parenting Styles and Dimensions Questionnaire (*n* = 1).

### 3.3 Summary of Findings From Observational Studies

#### 3.3.1 Caregiver Dietary Intakes, Diversity and Quality

Three articles from the Nutrition Collaborative Research Support Program longitudinal study in Egypt and Kenya in the mid‐1980s used naturalistic home observations of caregiver–child interactions and assessed caregiver dietary intakes. In the Kenyan cohort, Wachs et al. (1992) reported that greater caregiver dietary intakes of energy and macronutrients was associated with less physical contact with the child, whereas in the Egyptian cohort greater intakes were associated with fewer non‐verbal responses and more vocalisations towards the child, but slower speed of respond to children's distress signals (Wachs et al. 1992). The authors speculated that cultural factors may mediate this relationship (e.g., Egyptian toddlers spend more time inside dwellings close to caregivers compared to Kenyan toddlers). Interestingly, the children's dietary intakes explained more variation in caregiving behaviours than caregivers' dietary intakes. In a second article, poor diet quality among Egyptian caregivers was associated with reduced responsiveness to infant vocalisations (Rahmanifar et al. 1993). Additionally, smaller, less vocal and less alert infants received less vocalisations from their caregivers. Finally, Azizi‐Egrari et al. (2004) found no associations between caregiver dietary intakes and caregiving behaviours in Kenya (although a different coding scheme was used for assessing caregiving behaviours), but caregivers did spend more time caring for and holding lower birth weight infants compared to higher birth weight infants (Azizi‐Egrari et al. 2004). Findings from these articles suggest that simultaneous consideration of both caregiver and child nutrition may be crucial for understanding the processes by which nutrition influences caregiving behaviours.

During a temporary food shortage in Kenya, reductions in caregiver energy intake altered caregiving behaviours, with the most significant impact among the poorest families (McDonald et al. 1994). Caregivers spent less time holding/carrying and caring for their children during the 3.5‐month food shortage. Mothers from families with higher socioeconomic backgrounds maintained a higher percentage of their energy intakes during the food shortage; however, a reduction in the amount a mother verbalised to and cared for her child compared to other caregivers suggested that alternative caregivers assumed caregiving responsibilities during the food shortage.

Greater caregiver dietary diversity was associated with higher stimulation scores using the FCI tool among children < 2 years of age in Malawi (Bliznashka et al. 2024). A longitudinal study in Canada reported a statistically significant interaction between maternal diet quality during pregnancy (determined by the Healthy Eating Index 2010 at 16–21 weeks' gestation) and HOME scores on measures of child executive function at 3–4 years of age, suggesting that a healthier pregnancy diet could potentially benefit child neurodevelopment in households experiencing barriers to providing stimulation and early learning opportunities to children (Mortaji et al. 2021). Both studies adjusted for potential caregiver and household confounders.

#### 3.3.2 Caregiver Anthropometry

Several studies demonstrated positive associations between caregiver anthropometric measures and stimulation and caregiver interactions in LMICs. In Bangladesh, caregiver BMI and MUAC were significantly positively correlated with caregiving scores using the HOME Inventory (Hamadani et al. 2012). Caregiver BMI was predictive of better HOME scores in Malawi among caregivers and their children with severe acute malnutrition (Daniel et al. 2021). Caregiver BMI was not associated with household income, caregiver age or number of children in the home. Vietnamese caregivers' height and Bangladeshi caregivers' BMI were positively associated with stimulation practices using the FCI among children 24–59.9 months of age but not among children 6–23.9 months (adjusting for father's occupation, household wealth and number of children < 5 years in the household) (Basnet et al. 2020).

In contrast to the findings described above, naturalistic home observations of caregiver–child interactions demonstrated mixed findings between caregiver weight and caregiving behaviours. In Kenya, caregiver weight and BMI were not associated with caregiving behaviours (Azizi‐Egrari et al. 2004). However, a study in Chile found caregiver weight was positively associated with caregiver sensitivity (ability to perceive and promptly respond to the child's signals) (Valenzuela 1997). In addition to weight, caregiver height, education and marital satisfaction accounted for most of the variance in caregiver sensitivity, suggesting that the quality of a caregiver's health, education and social relationships influence a caregiver's ability to provide sensitive caregiving more than indicators of socioeconomic disadvantage (e.g., household income or size).

Two articles observed caregiver–child interactions in research laboratories. One study among a small number of caregivers in the USA (*n* = 4 caregivers with obesity and *n* = 3 with normal weight) reported that caregivers with obesity interacted less with their infants over a 24‐h observation than caregivers with normal weight (381 vs. 570 min, respectively) (Rising and Lifshitz 2005). Additionally, body weight and body fat composition negatively correlated with interaction time. In Germany, during a laboratory‐based free play interaction, caregivers with obesity demonstrated less sensitivity and less optimal structuring, and more intrusiveness and hostility compared to caregivers with normal weight on the Emotional Availability Scale (Bergmann et al. 2016). Caregivers with obesity reported higher levels of depressive symptoms, although there was no significant effect of depressive symptoms on emotional availability scores. However, the association between caregiver obesity and lower emotional availability scores was mediated by lower capabilities to understand emotions, suggesting caregivers with obesity may have misunderstood their child's cues. However, it should be noted in these studies that the unfamiliar surroundings of the research laboratory may have contributed to changes in the caregiver–child interaction compared to home settings. A naturalistic home observation of caregiver–child dyads also in Germany reported lower quality of caregiver–child attachment among caregivers with obesity compared to caregivers with normal weight matched by education level (Keitel‐Korndörfer et al. 2015).

#### 3.3.3 Caregiver Anaemia/Iron Status

Two articles utilising naturalistic home observations in LMICs found caregiver haemoglobin concentrations influenced caregiving behaviours with infants ≤ 6 months of age. Egyptian caregivers with lower haemoglobin concentrations were less responsive to infant vocalisations (Rahmanifar et al. 1993). In Kenya, caregivers with anaemia (haemoglobin < 12.5 g/dL) spent less time holding and caring for their infants than non‐anaemic caregivers (39% vs. 48% of their time, respectively) (Azizi‐Egrari et al. 2004). Caregiver anaemia was not associated with household size or socioeconomic status, infant weight gain or morbidity, suggesting these factors did not explain the reduced caregiving time. Conversely, there was no relationship between caregiver anaemia (haemoglobin < 11 g/dL) and HOME scores among mothers at 6–9 weeks postpartum in Bangladesh (Hamadani et al. 2012).

In HICs, caregivers with anaemia (haemoglobin < 12 g/dL) in Uruguay were less likely to encourage their children to play and explore their environment compared to caregivers without anaemia, adjusting for child age and sex, maternal depressive symptomology and household size and socioeconomic status (Kordas et al. 2011). Additionally, caregivers of children with anaemia (haemoglobin < 11 g/dL) scored lower on their perceptions of their emotional support and sensitivity to their child's needs than caregivers of children without anaemia (e.g., less likely to show affection or tolerance towards their child's negative feelings or less likely to stop what they were doing to attend to the child's needs). In a UK case–control study, no statistically significant differences in caregiver perception of mother–infant bonding were found between anaemic and non‐anaemic mothers within 4 weeks of delivery (Dearman et al. 2012). However, the small sample size (*n* = 57 anaemic mothers (haemoglobin < 10 g/dL) and *n* = 58 non‐anaemic mothers) may have lacked power to detect differences, and mothers in this hospital trust were strongly encouraged to breastfeed and bond with their infant. Finally, in the USA, significant interactions between iron status and depression in relation to authoritarian parenting style (low warmth, high punishment and directiveness) were found, independent of sociodemographic factors (caregiver education, employment and income) (Aubuchon‐Endsley et al. 2012). Among caregivers with haemoglobin < 14 g/dL, depressive symptoms were positively associated with self‐reported authoritarian parenting style. No interactions were found for other markers of iron status, namely serum ferritin and soluble transferrin receptor.

#### 3.3.4 Maternal Vitamin B6 Status

One article examined breastmilk vitamin B6 concentration as an indicator of maternal vitamin B6 status and observed caregiver–child interactions over the first 6 months postpartum in Egypt (McCullough et al. 1990). Poor maternal vitamin B6 status (breastmilk vitamin B6 concentration < 430 nmol/L), was associated with greater nonresponse to infant vocalisations, less effective intervention to infant distress and less time spent on childcare (e.g., greater utilisation of sibling caregivers). Maternal age, parity, socioeconomic status, anthropometry, food intake and iron status were not associated with caregiver–child interaction variables. Additionally, maternal vitamin B6 status was found to influence infant behaviour; infants of mothers with low vitamin B6 status were less fussy but more irritable and prone to intense distress when presented with increasingly aversive stimuli and less easily consoled by adults.

### 3.4 Summary of Findings From RCTs

#### 3.4.1 Iron Supplementation

Two articles from one RCT in South Africa reported mother–child interactions among anaemic and non‐anaemic postpartum women (Murray‐Kolb and Beard 2009; Perez et al. 2005). Mothers with iron deficiency anaemia were randomised to receive daily iron supplements (125 mg iron, 25 mg vitamin C, 10 μg folic acid) or placebo (25 mg vitamin C, 10 μg folic acid) from 10 weeks postpartum to 9 months postpartum. A control group of non‐anaemic women received no supplement. Mother–child interactions during free play were videotaped at a health clinic and coded using either the Parent/Caregiver Involvement Scale (Perez et al. 2005) or the Emotional Availability Scale (Murray‐Kolb and Beard 2009). At baseline, anaemic mothers verbalised less, were less responsive and exerted more control over their infant's behaviour. At follow‐up, anaemic mothers treated with iron and non‐anaemic control mothers scored significantly higher on the sensitivity, structuring, responsiveness and non‐hostility scales than the anaemic placebo group (Murray‐Kolb and Beard 2009), while the anaemic placebo group demonstrated negative statements and lower responsiveness (Perez et al. 2005). These findings suggest that iron deficiency anaemia alters mother–child interactions and that these alterations are responsive to iron therapy.

#### 3.4.2 Multiple Micronutrient Supplementation

Two articles reported no effect of multiple micronutrient supplementation (MMS) on caregiving behaviours. In Bangladesh, daily supplementation from early pregnancy to 3 months postpartum did not improve the quality of mother–child interaction scores on the Nursing Child Assessment Satellite Testing Feeding Scale (NCAST) during a home‐based feeding interaction compared to 30 mg iron supplementation (Frith et al. 2009). Notably, women supplemented with a higher dose of iron (60 mg daily) had lower interaction scores and exhibited less positive and more negative interaction behaviours than the other two groups. A RCT in Malawi found no differences in HOME scores at child age 6 months between women supplemented with daily iron‐folic acid, MMS or small‐quantity lipid‐based nutrient supplements from pregnancy to 6 months postpartum (Prado et al. 2018). Additionally, HOME scores were not associated with biomarkers of maternal nutritional status, including haemoglobin, plasma retinol and breastmilk concentrations of multiple B vitamins and docosahexaenoic acid (DHA).

#### 3.4.3 Fish Oil Supplementation

Among Bangladeshi women supplemented with fish oil (containing DHA) and soy oil (containing linolenic acid and α‐linolenic acid) capsules daily from pregnancy to delivery, there was no difference in stimulation and caregiver interactions between the two groups at 10 months postpartum, assessed using the HOME Inventory (Tofail et al. 2006).

#### 3.4.4 Prenatal Food‐Based Supplement

A RCT in Bangladesh evaluated the impact of food insecurity and early versus later (~9 vs. ~20 weeks' gestation) initiation of a prenatal food‐based snack supplement on mother–child interaction (Frith et al. 2012). In the late initiation group, food insecurity was associated with lower interaction scores on the NCAST during a feeding interaction at child age 3–4 months. Conversely, food insecurity was not associated with reduced quality mother–child interaction among those assigned to the early initiation of the food supplement. The lowest quality interaction was found among severely food‐insecure dyads who were assigned to the late initiation group. It should be noted that the food supplement was administered by nutrition educators, providing the early initiation group with increased social contact and social support, though this was not measured.

## 4 Discussion

This scoping review summarises the available evidence on broad measures of caregiver nutrition and two nurturing care components, responsive caregiving and early learning, which are critical for optimal early childhood development. Existing literature was heterogeneous and included diverse caregiver nutrition assessments and employed various caregiver–child interaction assessment methodologies and tools. The available literature indicates that less optimal caregiver dietary intakes/quality, food insecurity, caregiver under‐ or overnutrition, anaemia and poor vitamin B6 status were associated with less responsive caregiving and less engagement in opportunities for early learning. Evidence available from a limited number of intervention studies (6 articles reporting on 4 unique RCTs) indicated that iron repletion of anaemic mothers or providing food‐based snacks to food insecure pregnantmothers positively altered caregiver–child interactions, but supplementation of caregivers who were not specifically undernourished with macronutrients or multiple micronutrients did not influence caregiving behaviours.

Observational evidence consistently demonstrated that various measures of caregiver nutrition (dietary intakes/quality, anthropometry, micronutrient status) were associated with more optimal caregiving in the form of responsive caregiving and engagement in stimulating early learning experiences independent of caregiver and household factors known to influence caregiving (e.g., caregiver age and education; household wealth and size). Caregivers' nutrition may influence caregiving behaviours by affecting their cognitive functioning, mood and physical capacity. Macro‐ and micronutrients (e.g., vitamins B6 and B12, folate, iron) play critical roles in the synthesis, metabolism and regulation of neurotransmitters responsible for cognitive and emotional functioning and behaviour (Rechenberg and Humphries 2013). Nutrient supplementation among pregnant and postpartum women has been shown to improve cognition and mood, promoting cognitive skills such as reasoning, decision‐making, learning and processing new information and memory (Beard et al. 2005; Prado et al. 2018; Prado et al. 2012). Consequently, increased cognitive capacity of caregivers may enable more effective care for young children. This is supported by findings that higher cognitive capacity, independent of education and household socioeconomic characteristics, is associated with improved caregiving behaviours, including higher quality child dietary intake (Bhargava and Fox‐Kean 2003; Wachs et al. 2005) and greater stimulation within the home (Baker‐Henningham et al. 2003). Furthermore, anaemia and depleted iron stores are associated with caregiver fatigue, poor mental health (stress, anxiety and depression) and negatively impact caregiver quality of life (Beard et al. 2005; Moya et al. 2022), which likely impacts caregiver–child interactions. Future research should design studies to further elucidate the pathways and mechanisms through which caregiver nutrition impacts specific caregiving behaviours. Furthermore, it is currently unknown if suboptimal caregiver nutrition influences caregiving behaviours in the context of parenting programmes that target these behaviours (i.e., responsive caregiving and stimulation). Delineating these mechanisms may provide insights into potential impact pathways of multicomponent interventions that aim to promote early childhood development through supporting caregiver nutrition and caregiving behaviours.

Children's dietary intakes and nutritional status additionally, and sometimes more strongly, influenced caregiving behaviours, although limited studies simultaneously considered these factors (Azizi‐Egrari et al. 2004; Rahmanifar et al. 1993; Wachs et al. 1992). This supports the functional isolation hypothesis, which proposes that undernourished children exhibit reduced activity, decreased environmental exploration and diminished ability to seek and receive stimulating interactions with caregivers (Levitsky and Barnes 1972). However, Kordas and colleagues observed that anaemic caregivers were less likely to encourage children to explore their environment, suggesting that this relationship may not be unidirectional, and that caregiver nutrition may partially influence these caregiving decisions. Future studies should explore the complex relationship between child and caregiver nutrition and caregiving and the reasoning behind such caregiver decisions. Interventions aimed at promoting child development may benefit from this insight and consequently more effectively support caregivers in providing nurturing care to their children.

Findings from the limited number of intervention trials were mixed. Two potential explanations may account for this observation. First, iron repletion of anaemic mothers and providing caregivers experiencing food insecurity and chronic undernourishment with earlier access to a supplementary food yielded positive impacts on caregiver–child interactions (Frith et al. 2012; Murray‐Kolb and Beard 2009; Perez et al. 2005). This aligns with studies demonstrating positive impacts of micronutrient supplementation on caregiver cognitive function among undernourished and anaemic women (Beard et al. 2005; Prado et al. 2012) and with research indicating benefits of small‐quantity lipid‐based nutrient supplements on birth outcomes among women with a BMI < 20 kg/m^2^ and those experiencing greater food insecurity (Dewey et al. 2024). Conversely, the remaining supplementation trials did not specifically target undernourished caregivers. Moreover, high‐dose iron supplementation among Bangladeshi women negatively impacted the quality of interactions compared to a lower dose, possibly due to side effects of high‐dose iron supplementation or by increasing caregiver morbidity, particularly among iron‐replete women (Frith et al. 2009). Additional research specifically targeting undernourished caregivers is needed to understand how nutritional interventions can benefit caregiving behaviours.

Secondly, tools used to assess caregiving behaviours may explain inconsistent findings from intervention studies. In this scoping review, we focused on outcomes broadly related to two Nurturing Care Framework components, responsive caregiving and early learning. Consistent with a recent review of measurement tools for assessing nurturing care (Jeong et al. 2022), we found variability with regard to the tools used and the degree to which these tools measured these constructs versus broader aspects of the caregiver–child relationship. The HOME Inventory is a tool commonly used across settings to measure caregiving behaviours across six domains and broadly encompasses both responsive caregiving and early learning. However, in most cases, as with the two trials reporting null findings in this scoping review (Prado et al. 2018; Tofail et al. 2006), an aggregated total HOME score is reported as a single indicator representing both components of nurturing care. Therefore, the multidimensional HOME Inventory may not be sufficiently sensitive to detect subtle changes in caregiver behaviours in response to nutrient supplementation. Conversely, trials reporting positive impacts of supplementation on caregiver–child interaction used direct observations of interactions with extensive coding schemes (Frith et al. 2012; Murray‐Kolb and Beard 2009; Perez et al. 2005). However, these studies used tools that broadly measure some level of caregiver responsivity (e.g., Parent/Caregiver Involvement Scale) or the caregiver–child relationship or general caregiving qualities more broadly (e.g., NCAST, caregiver–child interactions) rather than responsive caregiving per se, due to a lack of validated tools that measure responsive caregiving consistent with the conceptual definition (Hentschel et al. 2024; Jeong et al. 2022). Future research on caregiver nutrition and nurturing care, particularly trials on nutrient supplementation and responsive caregiving, should carefully consider the use of direct observation tools specifically designed to measure responsive caregiving. Recent efforts to develop and validate separate Responsive Care (Hentschel et al. 2024) and Early Learning (Hentschel et al. 2024) tools will support this future research endeavour.

Limitations of the available evidence include largely observational studies, old data and small sample sizes. Notably, a third of the included articles comprised of data collected > 25 years ago, including five articles with data collected in the 1980s. Caregiver nutrition measures largely focused on dietary intakes/quality and anthropometry. Limited studies considered micronutrient deficiencies beyond anaemia/iron deficiency. Deficiencies in vitamins B6 and B12, zinc and magnesium can lead to mood disturbances, cognitive impairments and behavioural changes. Evidence has linked suboptimal levels of fatty acids and micronutrients (e.g., folate, vitamin D, zinc) with an increased risk of perinatal depression (Sparling et al. 2017), which has been associated with negative caregiving practices (Huang et al. 2017; Scherer et al. 2019). Limited studies considered how caregiver mental well‐being may mediate the relationship between nutrition and caregiving behaviours. Caregiver behaviours and responses towards children vary across cultural settings. For example, non‐Western caregivers may use few distal (e.g., vocalisations, smiles) and more proximal (e.g., touch, carrying) responses (Kärtner et al. 2008). Tools to assess caregiving behaviours developed in Western settings may not maintain their validity in LMIC settings (Bozicevic et al. 2024). The selection or adaptation of tools that consider these cultural behaviours and norms is critical. Notably, articles included in this scoping review that assessed caregiving behaviours in LMICs reported using an adapted HOME Inventory for the local context or coding schemes that were validated for the country in which they were being used (e.g., the NCAST coding scheme in Bangladesh).

Limitations of this review include restrictions of articles published in English and only one database was searched, consistent with scoping review methodologies. Therefore, potentially relevant literature could have been missed. However, a scoping review provided a more appropriate methodology to examine a body of literature that has not been previously reviewed and included broad measures of caregiver nutrition (e.g., diet, anthropometry, micronutrient status). Furthermore, we included articles identified through other sources such as citation searching and the authors' knowledge. Likewise, consistent with scoping review methodologies, we did not conduct a quality assessment of the included articles. As our objective was to identify avenues for further research rather than make policy or programme recommendations, a formal quality assessment was not required. Future work could expand this scoping review by searching additional databases, including non‐English literature and conducting a quality assessment in a more in‐depth systematic review of the topic.

## 5 Conclusion

This scoping review summarises available published evidence on broad measures of caregiver nutrition and responsive caregiving and early learning behaviours, identifying knowledge gaps and areas for future research. Existing literature identified consistent associations between less optimal dietary intakes, caregiver under‐ and overnutrition and anaemia with less responsive caregiving and engagement in early learning from observational studies. Evidence for micronutrients other than anaemia/iron deficiency was limited. The small number of RCTs identified revealed inconsistent findings but suggests nutritional interventions targeting undernourished caregivers could potentially have a positive impact on caregiving behaviours. Further research is required, particularly from trials that specifically target undernourished caregivers and a wider range of macro‐ and micronutrients. Consideration should be given to caregiver psychosocial well‐being (e.g., stress, anxiety, depression), which may influence the association between caregiver nutrition and caregiving behaviours. Direct observation tools specifically designed to measure responsive caregiving may be less prone to bias and more sensitive than multidimensional or self‐reported measures in detecting changes in caregiving behaviours, especially in intervention studies. Future research should explore multicomponent child development interventions and programmes that combine nutritional support for both caregivers and children, behavioural recommendations for responsive care and stimulation and mental well‐being support for caregivers.

## Author Contributions

Taryn J. Smith conceptualised the study and performed the search. Taryn J. Smith and Alice Fortune screened the articles and extracted data. Taryn J. Smith drafted the manuscript. All authors contributed intellectually and critically reviewed the manuscript. All authors read and approved the final version of the manuscript.

## Conflicts of Interest

The authors declare no conflicts of interest.

## Supporting information


**Table S1.** Medline (Ovid) search strategy.
**Table S2.** Inclusion and exclusion criteria.

## Data Availability

Data sharing does not apply to this article as no data sets were generated or analysed during the study.
