# Supplementary material for: Caregiver Nutrition and Nurturing Care: A Scoping Review
Source: Matern Child Nutr. 2025 Jun 16;21(4):e70058. doi: 10.1111/mcn.70058 (PMC12454204; doi:10.1111/mcn.70058)
Supplement: Supplementary file 1 — Table S1. Medline (Ovid) search strategy. Table S2. Inclusion and exclusion criteria. [file MCN-21-e70058-s001.docx]

**Table S1.** Medline (Ovid) search strategy

| **Domain** | **Search terms** |
| --- | --- |
| #1  Population group | Pregnancy/ OR Postpartum Period/ OR Mothers/ OR Lactation/ OR Breast Feeding/ OR pregnan* OR postpartum OR post-partum OR maternal OR mother* OR lactation OR breastfeed* OR “breast feed*” |
| #2  Nutrition (exposure) | Nutritional Status/ OR Nutrients/ OR Malnutrition/ OR Micronutrients/ OR Body Mass index/ OR Anemia/ OR Anemia, Iron Deficiency/ OR Diet/ OR Food/ or Dietary Supplements/ OR nutrition* OR nutrient* OR malnutrition OR malnourish* OR undernutrition OR undernourish* OR micronutrient* vitamin* OR mineral* OR anthropom* OR anaemia OR anemia OR anaemic OR anemic OR diet* OR food* OR “dietary intake” OR “dietary diversity” OR “food intake” OR energy OR supplement* |
| #3  Caregiving (outcome) | Mother-Child Relations/ OR Parent-Child Relations/ OR Maternal Behavior/ OR “caregiver infant interaction” OR “caregiver child interaction” OR “maternal infant interaction” OR “maternal child interaction” OR “mother infant interaction” OR “mother child interaction” OR “parent infant interaction” OR “parent child interaction” OR “maternal behav*” OR “caregiver behav*” OR “family care behav*” OR “maternal sensitivity” OR “parental sensitivity” OR “responsive caregiving” OR “responsive parenting” OR “early learning” OR “psychosocial stimulation” OR “responsive stimulation” OR “home environment” |
| Search strategy | (#1 adj2 #2) AND #3 |

**Table S2.** Inclusion and exclusion criteria

|  | **Inclusion criteria** | **Exclusion criteria** |
| --- | --- | --- |
| **Population** | Pregnant women, postpartum women, mothers of young children <5 years of age, mother-child dyads where the child is <5 years of age.  Human studies. | Mothers or mother-child dyads where the child is >5 years of age, studies that include fathers only or couples only with no maternal measures.  Animal studies. |
| **Exposure** | Any measure of maternal anthropometry, biomarkers of nutritional status, maternal dietary intakes or indicators (e.g. dietary diversity), or any intervention that provided pregnant or postpartum women with macro- or micronutrient or food supplements or fortified foods. | No measure of maternal nutritional status or dietary intakes/indicators or no nutrient intervention. |
| **Outcomes** | Outcome measures of psychosocial caregiving, including maternal-child interactions/relationships, responsive caregiving, stimulation and opportunities for early learning in the home. | No outcomes related to caregiving, or only reporting maternal cognition, mood or mental health outcomes. |
| **Study design** | Cross-sectional studies, cohort studies, randomised controlled trials and controlled before-after studies. | Case studies, systematic or other review articles, conference abstracts, opinions, editorials, commentaries, protocols and qualitative studies. |
